# Long-term effects of safinamide adjunct therapy on levodopa-induced dyskinesia in Parkinson’s disease: post-hoc analysis of a Japanese phase III study

**DOI:** 10.1007/s00702-022-02532-2

**Published:** 2022-08-24

**Authors:** Nobutaka Hattori, Takanori Kamei, Takayuki Ishida, Ippei Suzuki, Masahiro Nomoto, Yoshio Tsuboi

**Affiliations:** 1 Department of Neurology, Juntendo University School of Medicine, 2-1-1 Hongo, Bunkyo-ku, Tokyo, 113-8431 Japan; 2 Medical Headquarters, Eisai Co., Ltd., 4-6-10 Koishikawa, Bunkyo-ku, Tokyo, 112-8088 Japan; 3 Medicine Development, Deep Human Biology Learning, Eisai Co., Ltd., 4-6-10 Koishikawa, Bunkyo-ku, Tokyo, 112-8088 Japan; 4 Saiseikai Imabari Center for Health and Welfare, 7-6-1 Kitamura, Imabari, Ehime 799-1592 Japan; 5 Department of Neurology, Fukuoka University, 7-45-1 Nanakuma, Jonan-ku, Fukuoka, 814-0180 Japan

**Keywords:** Safinamide, Clinical trial, Post hoc analysis, Dyskinesia, MAO-B inhibitor, Parkinson’s disease

## Abstract

This post-hoc analysis investigated the long-term effects of safinamide on the course of dyskinesia and efficacy outcomes using data from a phase III, open-label 52-week study of safinamide 50 or 100 mg/day in Japanese patients with Parkinson’s disease (PD) with wearing-off. Patients (*N* = 194) were grouped using the UPDRS Part IV item 32: with and without pre-existing dyskinesia (pre-D subgroup; item 32 > 0 at baseline [*n* = 81], without pre-D subgroup; item 32 = 0 at baseline [*n* = 113]). ON-time with troublesome dyskinesia (ON-TD) increased significantly from baseline to Week 4 in the pre-D subgroup (+ 0.25 ± 0.11 h [mean ± SE], *p* = 0.0355) but gradually decreased up to Week 52 (change from baseline: − 0.08 ± 0.17 h, *p* = 0.6224); ON-TD did not change significantly in the Without pre-D subgroup. UPDRS Part IV item 32 score increased significantly at Week 52 compared with baseline in the Without pre-D subgroup, but no UPDRS Part IV dyskinesia related-domains changed in the pre-D subgroup. Both subgroups improved in ON-time without TD, UPDRS Part III, and Part II [OFF-phase] scores. The cumulative incidence of new or worsening dyskinesia (adverse drug reaction) at Week 52 was 32.5 and 5.0% in the pre-D and Without pre-D subgroups, respectively. This study suggested that safinamide led to short-term increasing dyskinesia but may be not associated with marked dyskinesia at 1-year follow-up in patients with pre-existing dyskinesia, and that it improved motor symptoms regardless of the presence or absence of dyskinesia at baseline. Further studies are warranted to investigate this association in more details.

Trial registration: JapicCTI-153057 (Registered: 2015/11/02).

**Supplementary Information:**

The online version contains supplementary material available at 10.1007/s00702-022-02532-2.

## Introduction

Levodopa has yet to be surpassed as the most effective oral treatment for motor symptom control in Parkinson’s disease (PD) (Armstrong and Okun 2020; LeWitt and Fahn 2016). Motor complications such as involuntary movements (i.e., dyskinesias) and wearing-off often occur in PD as the disease progresses and within an estimated 6.5 years of chronic treatment with levodopa (Tran et al. 2018). Such dyskinesias can lead to impaired activities of daily living (ADL) (Pahwa et al. 2019) and quality of life (QoL) (Montel et al. 2009; Péchevis et al. 2005).

In addition to a reduction in levodopa dose, a dose reduction or discontinuation of monoamine oxidase (MAO)-B inhibitors or other dopaminergic drugs may be considered for patients with advanced PD who develop troublesome dyskinesia. As such, it is often difficult to choose a treatment that improves wearing-off without the patient newly developing dyskinesia or aggravating pre-existing dyskinesias (Hinson 2010).

The development of levodopa-induced dyskinesia (LID) is attributed to a decrease in dopamine neurons or stimulation of D1 receptors upon intermittent stimulation of direct pathway striatal neurons by levodopa (Cenci and Lundblad 2006; Ding et al. 2015). Furthermore, both animal and clinical studies have revealed that LID is linked to increases in extracellular glutamate. However, the exact pathophysiological mechanisms driving LID are still elusive, and those mentioned here are among many possible speculations.

Safinamide is a selective and reversible MAO-B inhibitor approved as an add-on therapy for patients with PD who are experiencing motor fluctuations with levodopa (Kurihara et al. 2021). In addition to MAO-B inhibition, safinamide modulates glutamate release by inhibiting sodium channels in vivo (Morari et al. 2018). In a preclinical study using a primate model of macaque monkeys, safinamide suppressed LID but increased the period of antiparkinsonian response to levodopa (Grégoire et al. 2013).

Several clinical studies have examined the effects of safinamide on dyskinesias, but the findings have been controversial. Study 018 reported that the change in the Dyskinesia Rating Scale was not significantly different between the safinamide and placebo groups after 24 months of treatment. However, in a subpopulation with moderate or severe dyskinesia, there was an improvement in the Dyskinesia Rating Scale with safinamide 100 mg/day (Borgohain et al. 2014; Cattaneo et al. 2015). In a large European observational study (SYNAPSES) in PD patients with wearing-off, the proportion of patients with dyskinesia tended to decrease from baseline (from 39.2 to 27.8%) after 12 months’ administration of safinamide 50 mg/day or 100 mg/day; however, in 13.7% of patients, dyskinesia was reported as a common adverse event (AE) after safinamide administration (Abbruzzese et al. 2021).

Although there have been long-term studies conducted to assess the effect of safinamide on dyskinesia after 24 months (Borgohain et al. 2014; Cattaneo et al. 2015), none of these reports analyzed the effects of safinamide on dyskinesia over time. Therefore, we considered it meaningful to explore the profile of safinamide according to the presence/absence of dyskinesia for treatment selection in a clinical setting. Thus, in this post-hoc study, we aimed to investigate the long-term effect of safinamide on the course of dyskinesia using the results of a Japanese phase III 52-week study of safinamide 50 mg/day or 100 mg/day in Japanese patients with PD who had wearing-off (Tsuboi et al. 2020). We also explored the safety and efficacy of safinamide for patients with dyskinesia prior to administration (pre-D group), as well as the incidence rate of new-onset dyskinesia.

## Methods

### Study design

The study was a phase III, multicenter, open-label study conducted at 29 centers in Japan between December 2015 and November 2017. Details of the study design are published elsewhere (Tsuboi et al. 2020). Briefly, the study included a 4-week observation (screening) period and a 52-week treatment period (4 weekly follow-ups from baseline [Visit 2] to the end of study drug administration or drug discontinuation [Visit 15]; Online Resource 1).

The study protocol was approved by an ethics committee at each study site, and all patients provided informed consent before study initiation. The study conduct adhered to the ethical principles of the Declaration of Helsinki, Good Clinical Practice Guidelines, and local laws and regulations. The study was registered in the Japan Pharmaceutical Information Center under the identifier JapicCTI-153057.

### Patients

The study’s target population included Japanese patients aged ≥ 30 years diagnosed with PD according to the UK Parkinson’s Disease Society Brain Bank diagnostic criteria. All patients received a levodopa combination drug with a stable dose regimen (three doses/day or more and 300 mg/day or more) and must not have started treatment with an anti-PD drug other than levodopa combination drugs or must not have undergone a change in the dose regimen of such therapy during the observation period. Additionally, patients were required to have a mean daily OFF time of 2 or more hours. All patients had a modified Hoehn and Yahr stage of II–IV during the OFF-phase. Patients with evidence of dementia, major psychiatric illnesses, and/or severe and progressive medical illnesses, as well as those receiving antipsychotics, antidepressants, or drugs with antagonistic dopamine action, were excluded.

### Treatment

Patients were administered safinamide at a dosage of 50 mg/day; the dose could be increased up to 100 mg once daily from Week 4 onward based on the following criteria: (1) there was no safety concern; (2) the therapeutic response to 50 mg/day was poor; and (3) the patient wanted to increase the dose. The criteria for dose reduction were as follows: (1) AEs attributable to excessive dopamine action made it difficult for the patient to continue the study; and (2) AEs did not improve even after reducing the dose of the levodopa combination drugs. A subsequent dose increase was prohibited.

### Clinical features associated with dyskinesia

The clinical features associated with dyskinesia were evaluated based on the change from baseline to Week 52 in the mean daily ON-time with troublesome dyskinesia (ON-TD), which was assessed using a 24-h symptom diary, and changes in the Unified Parkinson’s Disease Rating Scale (UPDRS) Part IV items 32 (duration of dyskinesia, score 0–4), 33 (severity of dyskinesia, score 0–4), or 34 (dyskinesia with pain, score 0–4) as evaluated by a physician. Physicians explained to the subjects how to complete the 24-h symptom diary until sufficient understanding was achieved. The cumulative incidence rate of dyskinesia as an adverse drug reaction (ADR) from baseline was also evaluated.

### Effects on wearing-off, motor symptoms, and ADL

The effects of wearing-off and motor symptoms were evaluated based on the change from baseline to Week 52 in the mean daily ON-time without troublesome dyskinesia (ON-WOTD), which was assessed using a 24-h symptom diary, and changes from baseline to Week 52 in UPDRS Part II (OFF-phase and ON-phase) and Part III (ON-phase).

### Statistical analysis

As this was a post-hoc analysis, statistical methods were not prespecified. Imputation for drop-out and missing data was not conducted. The significance level of all testing was 5% (two-tailed) and no adjustments were made for multiplicity. SAS version 9.4 (SAS Institute, Cary, NC, USA) was used for the statistical analysis.

The full analysis set (FAS) included all subjects who received at least one dose of the study drug, had available evaluations of ON-time at baseline, and had at least one follow-up evaluation. The safety analysis set consisted of all patients who received at least one dose of the study drug.

Categorical variables were summarized as frequencies using number (*n*) and percentage (%), and continuous variables were presented using summary statistics. Changes from baseline to each time point were summarized and compared using a paired *t* test with data from patients who had both baseline and post-baseline time point evaluations.

For subgroup analyses, patients were categorized into two groups based on the UPDRS Part IV item 32: those with pre-existing dyskinesia (pre-D; UPDRS Part IV item 32 > 0 at baseline) and those without pre-existing dyskinesia (Without pre-D; UPDRS Part IV item 32 = 0 at baseline). To compare the differences in baseline values between the two subgroups, a Welch’s *t* test was used for continuous variables, a Fisher’s exact test was used for categorical variables, and a Wilcoxon rank-sum test was used to compare the number of concomitant non-levodopa antiparkinsonian drugs. Additional subgroup analyses were also conducted in patients who completed 52 weeks of treatment without any changes in the dose of levodopa combination drugs.

## Results

### Patient characteristics

**Fig. 1:**
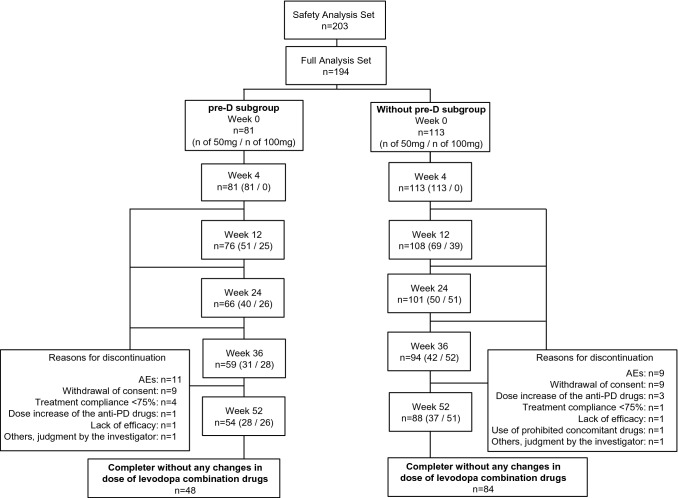
Flowchart showing patient disposition in this post-hoc analysis. Among AEs that led to discontinuation, dyskinesia was reported in 4 cases in the pre-D subgroup and 0 cases in the Without pre-D subgroup. All other AEs had different symptoms. *AE* adverse event, *PD* Parkinson’s disease, *pre-D* pre-existing dyskinesia *W* week

A total of 194 patients were included in the FAS, 132 patients completed the 52-week treatment without any changes in dose of levodopa combination drugs, and 203 were included in the safety analysis set (Fig. 1). Compared with the Without pre-D subgroup, the pre-D subgroup had a significantly higher proportion of women (75.3 vs 47.8%, *p* = 0.0001), significantly longer mean [standard deviation] duration of levodopa treatment (9.58 [4.41] years vs 5.44 [3.93] years, *p* < 0.0001), significantly higher levodopa doses (508.64 [183.34] mg vs 403.54 [129.04] mg, *p* < 0.0001), and significantly lower ADL according to the UPDRS Part II (OFF-phase) assessment (16.07 [6.86] vs 12.90 [7.71], *p* = 0.0029) at baseline (Table 1).

**Table 1:** Baseline characteristics of patients overall and in subgroups with and without pre-existing dyskinesia

	Safinamide 50/100 mg/day^a^			
	All	pre-D	Without pre-D	*p* value^b^ pre-D vs Without pre-D
	(*N* = 194)	(*N* = 81)	(*N* = 113)	
Sex, male (%)	79 (40.7)	20 (24.7)	59 (52.2)	0.0001
Age, years	67.17 (8.59)	66.28 (8.30)	67.81 (8.77)	0.2205
Duration of Parkinson’s disease, years	9.78 (5.25)	12.25 (5.59)	8.01 (4.19)	< 0.0001
Duration of treatment with levodopa, years	7.17 (4.60)	9.58 (4.41)	5.44 (3.93)	< 0.0001
Duration of wearing-off phenomenon, years	3.72 (3.13)	5.39 (3.41)	2.52 (2.27)	< 0.0001
Modified Hoehn and Yahr stage (ON-phase)	2.36 (0.72)	2.37 (0.72)	2.35 (0.72)	0.8099
Modified Hoehn and Yahr stage (OFF-phase)	3.34 (0.67)	3.54 (0.56)	3.19 (0.71)	0.0002
UPDRS part I	1.18 (1.60)	1.31 (1.43)	1.09 (1.71)	0.3311
UPDRS part II (ON-phase)	5.82 (5.18)	6.38 (5.19)	5.42 (5.15)	0.2010
UPDRS part II (OFF-phase)	14.23 (7.51)	16.07 (6.86)	12.90 (7.71)	0.0029
UPDRS part III (ON-phase)	21.27 (11.37)	20.28 (11.12)	21.98 (11.54)	0.3033
UPDRS part IV	4.97 (2.07)	6.44 (1.65)	3.91 (1.65)	< 0.0001
UPDRS items 32–34	0.98 (1.37)	2.30 (1.20)	0.04 (0.23)	< 0.0001
Mean daily ON-time with dyskinesia (non-troublesome and troublesome)	1.78 (3.04)	3.91 (3.49)	0.25 (1.29)	< 0.0001
Mean daily ON-TD, hours	0.34 (1.09)	0.76 (1.52)	0.04 (0.40)	< 0.0001
Mean daily ON-WOTD, hours	10.13 (2.77)	10.00 (2.68)	10.23 (2.83)	0.5817
Mean daily OFF-time, hours	5.87 (2.58)	5.71 (2.40)	5.98 (2.71)	0.4689
Dose of levodopa at baseline, mg	447.42 (162.16)	508.64 (183.34)	403.54 (129.04)	< 0.0001
Concomitant use of non-levodopa antiparkinsonian drugs, *n* (%)	180 (92.8)	80 (98.8)	100 (88.5)	0.0088
Dopamine agonists	159 (82.0)	74 (91.4)	85 (75.2)	0.0043
Entacapone	79 (40.7)	47 (58.0)	32 (28.3)	< 0.0001
Zonisamide	66 (34.0)	34 (42.0)	32 (28.3)	0.0648
Istradefylline	53 (27.3)	25 (30.9)	28 (24.8)	0.4144
Amantadine hydrochloride	54 (27.8)	31 (38.3)	23 (20.4)	0.0090
Trihexyphenidyl hydrochloride	28 (14.4)	16 (19.8)	12 (10.6)	0.0971
Droxidopa	7 (3.6)	2 (2.5)	5 (4.4)	0.7013

Data in the table are mean (SD), unless otherwise indicated

*AE* adverse event, *ON-TD* ON-time with troublesome dyskinesia, *ON-WOTD* ON-time without troublesome dyskinesia, *pre-D* pre-existing dyskinesia, *SD* standard deviation, *UPDRS* unified Parkinson’s disease rating scale

^a^The dose was increased from 50 mg/day to 100 mg/day from Week 4 if there were no safety concerns, the therapeutic response to 50 mg/day was poor, and the subject wanted to increase the dose. Subsequently, the dose could be decreased to 50 mg/day if AEs attributable to excessive dopamine action made it difficult to continue the study and AEs did not improve even after reducing the dose of the levodopa combination drug

^b^Differences in baseline values between two subgroups were compared using a Welch’s *t* test for continuous variables, a Fisher’s exact test for categorical variables, and a Wilcoxon rank-sum test for the numbers of concomitant non-levodopa antiparkinsonian drugs

### Clinical features associated with dyskinesia

**Fig. 2:**
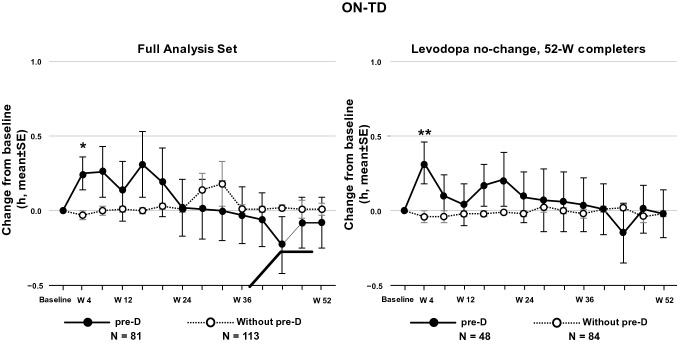
Average daily ON-time with troublesome dyskinesia (ON-TD) in the FAS. *FAS* full analysis set, *pre-D* pre-existing dyskinesia, *SE* standard error, *W* week. The *p* values indicate the difference at Week 4 vs baseline and were calculated using a paired *t* test based on patients who had both evaluations at baseline and each timepoint. *p = 0.0355; **p = 0.0246

In the pre-D subgroup, ON-TD increased significantly from baseline to Week 4 but gradually decreased up to Week 52 (Fig. 2). The mean (standard error) change from baseline to 52 weeks was − 0.08 (0.17) h (*p* = 0.6224). After 52 weeks, about half (FAS: 49%, Completed: 50%) of patients were taking the 100-mg/day dosage. In the Without pre-D subgroup, ON-TD did not change significantly compared with baseline throughout the observation period. A similar tendency was observed in the population who completed the study without any changes in the dose of levodopa combination drugs.

Regarding changes in UPDRS Part IV (Table 2), no significant changes were observed from baseline to Week 52 for items 32, 33, 34, or 32–34 together in the pre-D subgroup. However, the UPDRS Part IV item 32 score at Week 52 had significantly increased when compared with baseline in the Without pre-D subgroup.

**Table 2:** Changes from baseline to Week 52 in the UPDRS Part IV (FAS)

UPDRS Part IV		Safinamide 50/100 mg/day^a^							
		pre-D				Without pre-D			
		Item 32^b^	Item 33^c^	Item 34^d^	Items 32–34	Item 32^b^	Item 33^c^	Item 34^d^	Items 32–34
Baseline	*n*	81	81	81	81	113	113	113	113
	Mean (SE)	1.54 (0.08)	0.74 (0.08)	0.01 (0.01)	2.30 (0.13)	0.00 (0.00)	0.00 (0.00)	0.04 (0.02)	0.04 (0.02)
Week 52 (change from baseline)	*n*	54	54	54	54	88	88	88	88
	Mean (SE)	0.15 (0.13)	− 0.04 (0.13)	− 0.02 (0.02)	0.09 (0.19)	0.16 (0.05)	0.03 (0.03)	− 0.01 (0.01)	0.18 (0.06)
	*p* value vs baseline^e^	0.2712	0.7758	0.3219	0.6364	0.0014	0.1812	0.3201	0.0054

*AE* adverse event, *FAS* full analysis set, *pre-D* pre-existing dyskinesia, *SE* standard error, *UPDRS* unified Parkinson’s disease rating scale

^a^The dose was increased from 50 mg/day to 100 mg/day from Week 4 if there were no safety concerns, the therapeutic response to 50 mg/day was poor, and the subject wanted to increase the dose. Subsequently, the dose could be decreased to 50 mg/day if AEs attributable to excessive dopamine action made it difficult to continue the study and AEs did not improve even after reducing the dose of a levodopa combination drug

^b^Duration of dyskinesia

^c^Severity of dyskinesia

^d^Dyskinesia with pain

^e^The *p* value was calculated using a paired *t* test and was based on patients who had both baseline and Week 52 evaluations

**Fig. 3:**
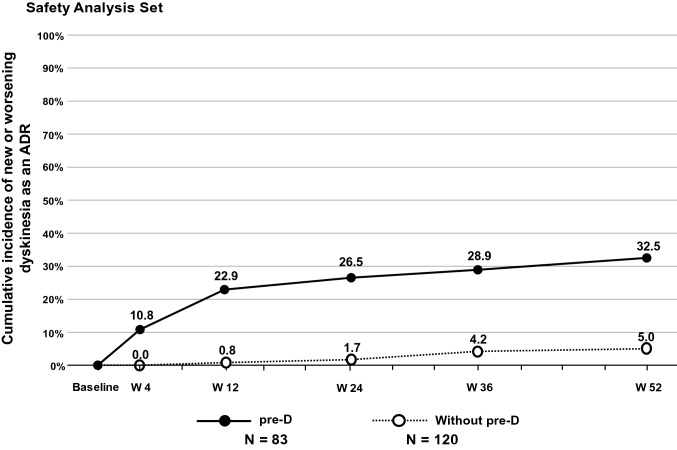
Progression of dyskinesia as an adverse drug reaction (safety analysis set). *ADR* adverse drug reaction, *pre-D* pre-existing dyskinesia, *W* week

The cumulative incidence of new or worsening dyskinesia as an ADR in both subgroups is shown in Fig. 3. The cumulative incidence of new or worsening dyskinesia as an ADR at Week 52 was lower in the Without pre-D subgroup (5.0%, 6 of 120 patients) compared with the pre-D subgroup (32.5%, 27 of 83 patients). In the pre-D subgroup, 19 of the 27 patients experienced dyskinesia as an ADR within 12 weeks.

### Effects on wearing-off, motor symptoms, and ADL

**Fig. 4:**
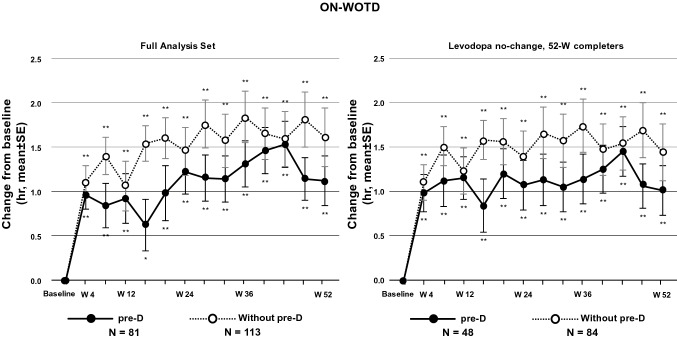
Average daily ON-time without troublesome dyskinesia (ON-WOTD) in the FAS. *FAS* full analysis set, *pre-D* pre-existing dyskinesia, *SE* standard error, *W* week. The *p* values indicate the difference from baseline and were calculated using a paired t-test based on patients who had both evaluations at baseline and each timepoint. *p < 0.05; **p < 0.01

ON-WOTD was significantly increased at each time points from Week 4 to Week 52 compared to baseline in both pre-D and Without pre-D patients (*p* < 0.05, each time point) (Fig. 4). A similar trend was observed in the population who completed the study without any changes in the dose of levodopa combination drugs.

Online Resource 2 shows the changes in UPDRS Part III, Part II (ON-phase), and Part II (OFF-phase). Both UPDRS Part II (OFF-phase) and Part III showed significant improvement (*p* < 0.01, each time point for both parts) from Week 4 to Week 52 compared to baseline, regardless of the presence of dyskinesia at baseline. UPDRS Part II (ON-phase) did not change from baseline in the pre-D subgroup, but improved significantly from Week 4 to Week 52 compared to baseline in the Without pre-D subgroup.

## Discussion

In this post-hoc analysis, safinamide temporarily worsened ON-TD, but this effect gradually eased during the 52-week treatment in the pre-D subgroup. An analysis of the subpopulation that completed 52 weeks of administration and did not undergo any changes in the dose of levodopa combination drugs showed that dyskinesia did not worsen in the long term, indicating that the long-term ON-TD reduction tendency did not result from patient drop-out or levodopa dose reduction. In the pre-D subgroup, none of the UPDRS Part IV dyskinesia related-domains worsened at Week 52 compared with baseline, which is consistent with the ON-TD result, and the incidence of new-onset dyskinesia reported as an ADR tended to be low. There were few cases of withdrawal due to AEs at the initial stage of safinamide administration. The number of dyskinesia cases that led to discontinuation of administration was small in both subgroups (*n* = 4) (Tsuboi et al. 2020). In addition, patients showed improvements in wearing-off, motor symptoms, and ADL (UPDRS Part II [OFF-phase]) regardless of the presence or absence of dyskinesia at baseline.

The definition of the pre-D subgroup was based on the clinician-reported UPDRS item 32 (duration of dyskinesia), with a score of 1 used as the cut-off. Of the 81 patients in the pre-D subgroup, 38 patients had troublesome dyskinesia at baseline (ON-TD > 0). In the pre-D subgroup, the proportion of women was higher, the duration of levodopa treatment was longer, and the dose of levodopa was higher than those in the Without pre-D subgroup, all of which are consistent with the reported risk factors for dyskinesia (Eusebi et al. 2018).

From the results of UPDRS Part IV, the duration of dyskinesia (item 32) was extended in the Without pre-D subgroup. In contrast, ON-TD was relatively stable, and UPDRS part II score (ON-phase) was significantly improved from baseline in this subgroup, which suggests that even though the time of dyskinesia increased, ADL were not impaired by this effect. However, the results of this study should be interpreted with caution because UPDRS Part IV and ADR were clinician-reported, while ON-TD and ON-WOTD were patient-reported using 24-h symptom diaries. In fact, a gap between physician assessment and patient self-awareness concerning the presence of dyskinesia has been recently reported (Ogura et al. 2021).

As safinamide inhibits the degradation of dopamine by inhibiting MAO-B (Kurihara et al. 2021) and an excess of dopamine causes dyskinesia, it is considered that a slight increase in ON-TD during the initial treatment phase is caused by the dopaminergic action of safinamide. Nonetheless, dyskinesia was not reported to worsen in the long term. Involvement of glutamate signaling has also been suggested in dyskinesias, given that glutamatergic striatal neurons are hypertrophied, and signal transduction is enhanced in the direct pathway (Cenci and Konradi 2010; Cenci and Lundblad 2006; Cerasa et al. 2014; Holtmaat and Svoboda 2009). Constitutive changes in the subunits of NMDA and AMPA receptors suggest an association with glutamate. In particular, it has been observed in animal and human studies that changes in the subunit of the NMDA receptor in the striatum are associated with the onset of dyskinesia (Gardoni et al. 2006, 2012; Mellone et al. 2015).

Safinamide has non-dopaminergic actions, such as its action as a sodium channel blocker, and it has been reported to suppress changes in the GluN2A/GluN2B ratio caused by chronic levodopa administration (Gardoni et al. 2018). Patients with LID have abnormal cortical facilitation, suggesting overactive glutamatergic neurotransmission in the cortex, and this dysfunction was restored via modulation of synaptic plasticity mechanisms by the long-term safinamide effect (Guerra et al. 2019, 2021). It is not clear whether this action of safinamide is mediated by blocking sodium channels; however, safinamide may affect dyskinesia. Studies have reported that amantadine, which has an NMDA receptor antagonistic effect, is effective against LID in the long term (Ory-Magne et al. 2014).

Because ON-TD may temporarily worsen after the administration of safinamide in the pre-D subgroup, it is essential to evaluate the location and time of dyskinesia carefully. During this study period, levodopa and other anti-PD drugs were prescribed at a set dosage, but it is necessary to consider adjusting the doses of these drugs during clinical practice.

LID has been reported to develop in approximately 36% of patients 4–6 years after levodopa initiation (Ahlskog and Muenter 2001). It affects more than 50% of PD patients who have been treated with levodopa for 5 years or more (Grandas et al. 1999). The baseline PD morbidity of the Without pre-D population in this study was 8.01 years, and the mean levodopa treatment duration was 5.44 years; therefore, the patient background suggested that this population was likely to develop LID. However, the cumulative incidence of dyskinesia at Week 52 was 5.0%, which is approximately 6 times lower than that in the pre-D subgroup (32.5%). The incidence of new AEs within 12 weeks of istradefylline treatment was reported to be 4.8% at 20 mg/day and 7.2% at 40 mg/day (Elmer et al. 2020); hence, the risk of new-onset ADRs with safinamide is not high. Nevertheless, the cumulative incidence of new or worsening dyskinesia as an ADR, which was relatively high in the Pre-D subgroup, warrants caution.

It does not seem likely that the long-term effects on dyskinesias were caused by reduced dopaminergic stimulation of safinamide resulting from the prolongation of ON-WOTD and the improvement of UPDRS Part II (OFF-phase) or Part III continued for 52 weeks in this study. The improvement of wearing-off with safinamide reportedly continued for up to 2 years (Borgohain et al. 2014; Tsuboi et al. 2020). The presence of dyskinesia at baseline indicates dopaminergic nerve degeneration and loss of dopamine buffering capacity; nonetheless, we observed that motor symptoms and wearing-off were also improved in the pre-D subgroup. Abnormal glutamate receptor activity in the basal ganglia has previously been reported in advanced PD (Cenci and Lindgren 2007; Espay et al. 2018; Gardoni et al. 2006; Sgambato-Faure and Cenci 2012), and as such, it is possible that the non-dopaminergic action of safinamide (Cattaneo et al. 2018) contributed to its efficacy in the pre-D subgroup.

### Limitations

The main limitations of this study were that it was a post-hoc analysis of an open-label, single-arm study, and statistical methods were not prespecified. Imputation for drop-out and missing data was not conducted, and no adjustments were made for multiplicity. It should be noted that few patients had troublesome dyskinesia at baseline in the pre-D group. Additionally, the treatment restrictions in this study are different from those in clinical practice. In clinical practice, measures such as increasing the number of doses of levodopa or reducing the daily dose should be considered. This interpretation of the FAS results is also limited because the dosage of safinamide and concomitant antiparkinsonian drugs could be adjusted at the onset of AEs, such as dyskinesia. Furthermore, the levodopa equivalent doses were not analyzed at each time point because data on concomitant antiparkinsonian drugs were often missing. Therefore, verification of our findings in clinical practice is also necessary. Finally, it should be noted that the definition of dyskinesia as an ADR includes onset and exacerbation of dyskinesia, but this distinction was not considered in this study.

## Conclusions

Regardless of the presence or absence of dyskinesia at baseline, long-term 52-week safinamide adjunctive treatment (50 and 100 mg/day) did not cause marked dyskinesia and improved ON-WOTD, motor symptoms, and ADL based on the UPDRS Part II (OFF-phase). Conversely, a short-term, mild increase in ON-TD was observed. This tendency was also observed in the limited population without any changes in the dose of levodopa combination drugs. In addition, the incidence of new-onset dyskinesia tended to be low. Regarding the precautions and benefits of safinamide in the pre-D subgroup at baseline, as with other anti-PD drugs, the addition of safinamide could be a treatment option for patients with wearing-off. However, further prospective clinical trials are warranted to investigate these potential benefits and the clinical relevance of safinamide on dyskinesia, for example, the difference in effects between safinamide 50 and 100 mg/day.

## Supplementary Information

Below is the link to the electronic supplementary material.

Supplementary file1 Study design *W* week (PDF 46 KB)

Supplementary file2 Change from baseline in UPDRS Part III, Part II (ON), and Part II (OFF) The *p* values indicate the difference from baseline. *p < 0.05; **p < 0.01 *Pre-D* pre-existing dyskinesia, *SE* standard error, *UPDRS* unified Parkinson’s disease rating scale, *W* week (PDF 89 KB)

## Data Availability

All data and materials support the reported claims and comply with standards of data transparency. Data will be made available on reasonable request.
