# Correction: Long-term effects of safinamide adjunct therapy on levodopa-induced dyskinesia in Parkinson’s disease: post-hoc analysis of a Japanese phase III study

**DOI:** 10.1007/s00702-022-02543-z

**Published:** 2022-09-08

**Authors:** Nobutaka Hattori, Takanori Kamei, Takayuki Ishida, Ippei Suzuki, Masahiro Nomoto, Yoshio Tsuboi

**Affiliations:** 1 Department of Neurology, Juntendo University School of Medicine, 2-1-1 Hongo, Bunkyo-ku, Tokyo, 113-8431 Japan; 2 Medical Headquarters, Eisai Co., Ltd., 4-6-10 Koishikawa, Bunkyo-ku, Tokyo, 112-8088 Japan; 3 Medicine Development, Deep Human Biology Learning, Eisai Co., Ltd., 4-6-10 Koishikawa, Bunkyo-ku, Tokyo, 112-8088 Japan; 4 Saiseikai Imabari Center for Health and Welfare, 7-6-1 Kitamura, Imabari, Ehime 799-1592 Japan; 5 Department of Neurology, Fukuoka University, 7-45-1 Nanakuma, Jonan-ku, Fukuoka, 814-0180 Japan

## Correction: Journal of Neural Transmission 10.1007/s00702-022-02532-2

The original version of this article unfortunately contained a mistake. Incorrect line in Figure 2.

The corrected Fig. 2 is given in the following page.

**Fig. 2:**
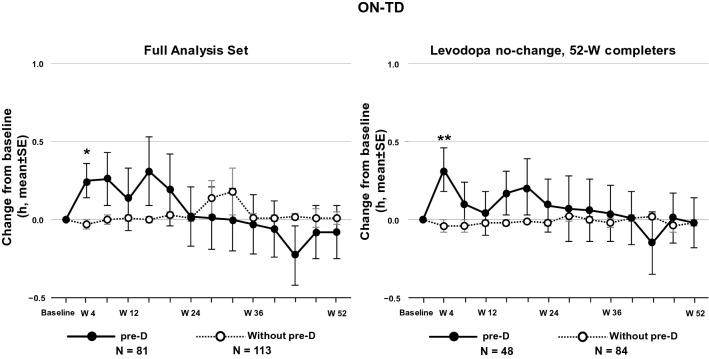
Average daily ON-time with troublesome dyskinesia (ON-TD) in the FAS. *FAS* full analysis set, *pre-D* pre-existing dyskinesia, *SE* standard error, *W* week. The *p* values indicate the difference at Week 4 vs baseline and were calculated using a paired *t* test based on patients who had both evaluations at baseline and each timepoint. *p = 0.0355; **p = 0.0246

