# Supplementary material for: Long-term effects of safinamide adjunct therapy on levodopa-induced dyskinesia in Parkinson’s disease: post-hoc analysis of a Japanese phase III study
Source: J Neural Transm (Vienna). 2022 Aug 24;129(10):1277–87. doi: 10.1007/s00702-022-02532-2 (PMC9468087; doi:10.1007/s00702-022-02532-2)
Supplement: Supplementary file 2 — Supplementary file2 Change from baseline in UPDRS Part III, Part II (ON), and Part II (OFF) The p values indicate the difference from baseline. *p < 0.05; **p < 0.01 Pre-D pre-existing dyskinesia, SE standard error, UPDRS unified Parkinson’s disease rating scale, W week (PDF 89 KB) [file 702_2022_2532_MOESM2_ESM.pdf]

Online Resource 2

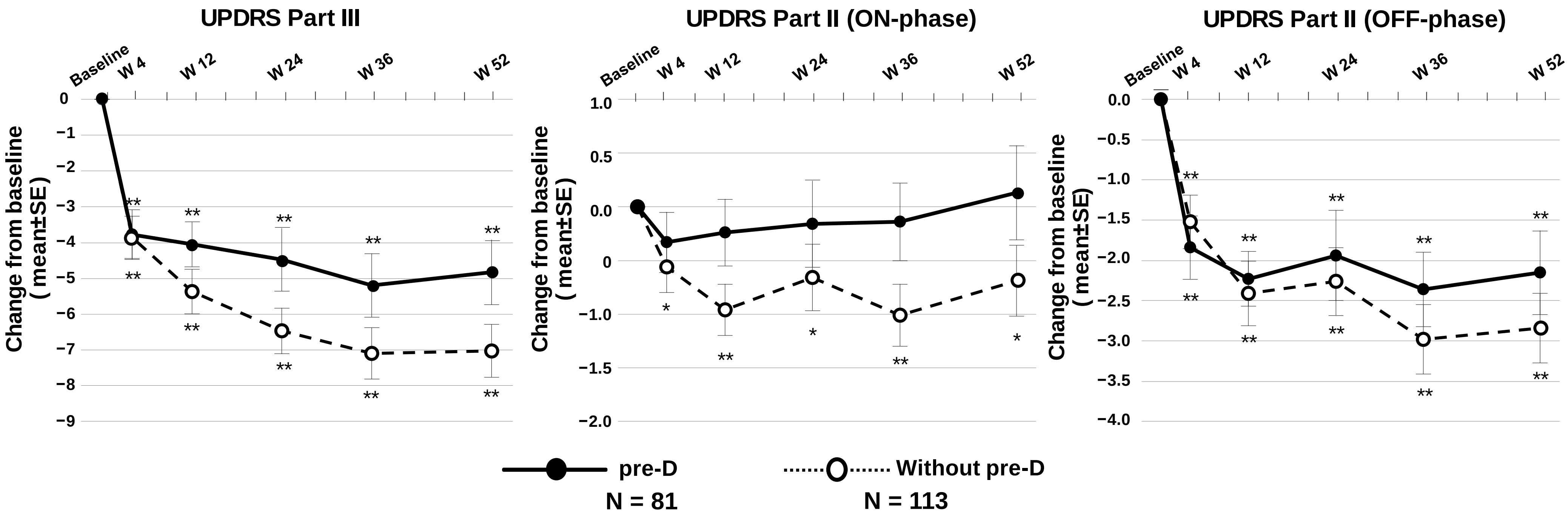

Long-term effects of safinamide adjunct therapy on levodopa-induced dyskinesia in Parkinson's disease: post-hoc analysis of a Japanese phase III study

Journal of Neural Transmission

Nobutaka Hattori*, Takanori Kamei, Takayuki Ishida, Ippei Suzuki, Masahiro Nomoto, Yoshio Tsuboi

*Corresponding author: Department of Neurology, Juntendo University School of Medicine, nhattori@juntendo.ac.jp
